# An ESR Framework for the Study of Consciousness

**DOI:** 10.3390/e23010097

**Published:** 2021-01-11

**Authors:** Diana Stanciu

**Affiliations:** 1 Romanian Young Academy, University of Bucharest, 050663 Bucharest, Romania; diana.stanciu@gmail.com; 2 Gerda Henkel Senior Research Fellow, Berlin Brandenburg Academy of Sciences and Humanities, D-10117 Berlin, Germany

**Keywords:** consciousness (generic/specific consciousness; phenomenal/access consciousness), free will (conscious free choice), neural correlates of consciousness (NCCs), global workspace theory (GWT), global neuronal workspace, integrated information theory (IIT), alterations of consciousness (AOCs), epilepsy, epistemic structural realism (ESR), ontic structural realism (OSR)

## Abstract

I will argue that, in an interdisciplinary study of consciousness, epistemic structural realism (ESR) can offer a feasible philosophical background for the study of consciousness and its associated neurophysiological phenomena in neuroscience and cognitive science while also taking into account the mathematical structures involved in this type of research. Applying the ESR principles also to the study of the neurophysiological phenomena associated with free will (or rather conscious free choice) and with various alterations of consciousness (AOCs) generated by various pathologies such as epilepsy would add explanatory value to the matter. This interdisciplinary approach would be in tune with Quine’s well known idea that philosophy is not simple conceptual analysis but is continuous with science and actually represents an abstract branch of the empirical research. The ESR could thus resonate with scientific models of consciousness such as the global neuronal workspace model (inspired by the global workspace theory—GWT) and the integrated information theory (IIT) model. While structural realism has already been employed in physics or biology, its application as a meta-theory contextualising and relating various scientific findings on consciousness is new indeed. Out of the two variants: ontic structural realism (OSR) and epistemic structural realism (ESR), the latter can be considered more suitable for the study of consciousness and its associated neurophysiological phenomena because it removes the pressure of the still unanswered ‘What is consciousness?’ ontological question and allows us to concentrate instead on the ‘What can we know about consciousness?’ epistemological question.

## 1. Introduction—The ESR as a Philosophical Framework for Studies of Consciousness in Neuroscience and Cognitive Science

Consciousness is a multifaceted phenomenon that can be approached from various directions: phenomenological, neurobiological, metaphysical, epistemological and cognitive [1]. Especially in contemporary neuroscience and cognitive science, consciousness studies seem to be in full swing. Despite the lack of a universally accepted operational definition and the criticisms of Gestalt psychology against the concept of consciousness as being only descriptive and not explanatory, specialists in neuroscience and cognitive science have recently made new discoveries on: consciousness and its neural correlates, consciousness and cognition in an evolutionary perspective, consciousness, empathy and their cognitive and affective components, the relationship between consciousness and higher brain functions such as free will and high-level perception, higher-order theories of consciousness (the analysis of conscious meta-mental states in terms of reflexive (meta-mental) self-awareness), and consciousness and embodied cognition [2,3,4,5,6,7,8,9].

All these themes related to consciousness are intensely debated issues of our society nowadays and to them we can add numerous others: the way we perceive and understand ‘reality’, the relevance of both reason and emotion in our conscious agency and decision-making (in both our personal lives and our social and political contexts), the way we try to ‘reproduce’ and ‘enhance’ reality through new technologies such as those involved in ‘virtual reality’ (VR), the way we try to build artificial consciousness, the way we consciously and empathically appreciate art as well as the degree in which we sometimes ‘lose’ the state of consciousness due to various pathological conditions.

In order to be precise about terminology, I would point out from the very beginning that I prefer the term ‘neurophysiological phenomena associated with consciousness’ to the well known ‘neural correlates of consciousness’ (NCCs) or the ‘neural basis of consciousness’ [10,11,12] when referring both to the study of consciousness and to its associated neurophysiological phenomena in general and to the study of particular cases such as that of free will (or conscious free choice) or that of various alterations of consciousness (AOCs) generated by epilepsy, for instance. This preference is meant to avoid any ambiguities the word ‘correlate’ may create in respect to the causal import and sufficiency of specific neurophysiological phenomena for consciousness, conscious free choice (and agency) or AOCs. What I particularly mean here is that I consider the causal relationship between the neurophysiological phenomena (that could mean brain configuration, electrical neuronal signal, chemical processes in the brain, etc.) and consciousness, conscious free choice and AOCs not as a sufficient and uni-directional line of causation, but rather as a multi-directional causal relation. While consciousness, conscious agency and AOCs may indeed appear as mental functions of the brain, it is not yet clear to what extent a specific brain area, electrical signal or chemical process can regularly be responsible for the same type of mental event in various contexts. The brain circuits and chemical processes form structural relations that are dynamic and still insufficiently studied and clarified. For a better understanding of these structural relations, we need to take into account neuroscience and cognitive science data processing in wider and more complex contexts.

Given all this complexity of aspects and research data, this article is a plea for interdisciplinary studies of consciousness that would offer a complex methodology and, consequently, complex results, with a higher explanatory value than an intently specialised study. This quest could be inspired by Quine’s idea that philosophy is not simple conceptual analysis, but the abstract branch of the empirical sciences [13] and by his attempt to understand science starting from its own resources while considering philosophy as continuous with science, as his well known statement: ‘philosophy of science is philosophy enough’ [14] suggests. I should also mention here the so-called ‘Quine-Putnam indispensability thesis’—the argument for the reality of mathematical entities—as a source of inspiration in this article since I consider that the mathematical expression of the empirical data would actually bridge any possible gap between science and philosophy. The indispensability thesis can be briefly summarised as follows: one must be committed to ‘all’ entities that are indispensable to our best scientific theories, and ‘only’ to those entities; mathematical entities are indispensable for our best scientific theories; consequently, one must be ontologically committed to mathematical entities [15].

Starting from these assumptions, I will attempt to prove that the epistemic structural realism (ESR) can offer a feasible philosophical meta-theory for the study of consciousness and its associated neurophysiological phenomena in neuroscience and cognitive science while also taking into account the mathematical structures involved in this type of research. This would be a new approach in the study of consciousness and I will argue for its advantages in what follows. In my view, the ESR would offer a feasible frame of reference for the processing of a multitude of data in neuroscience both on human consciousness in general and on specific aspects of it such as free will (conscious free choice) and AOCs generated by various pathologies—like epilepsy, for instance. These will be studied in more detail in Section 3 and Section 4 below. For the moment, I would just briefly point out that I consider ESR a philosophical meta-theory that could mediate between different mathematised theories of consciousness such as the global workspace theory (GWT) or the integrated information theory (IIT) while also encompassing network neuroscience models or, more generally, various structure-based scientific models. This meta-theory would only help contextualise, relate and bridge various existing scientific theories and models though. It will not offer a new scientific theory or model of consciousness. It would just propose a more complex and integrated meta-theoretic approach for the study of consciousness in general while relying on the principles of structural realism.

Structural realism has often been accepted as a feasible philosophical framework for science and especially for physics and biology [16,17,18,19,20,21,22,23,24,25,26,27]. For instance, starting from the external reality hypothesis, which postulates an external physical reality that is totally independent of human beings, Max Tegmark discussed the possibility of conceiving our physical world as an abstract mathematical structure (the mathematical universe hypothesis) provided that we use a sufficiently broad definition of mathematics [16]. In philosophical terms, what Tegmark might have referred to is one of the two versions of structural realism—the ontic structural realism (OSR)—which perfectly fits his study in physics.

But the OSR drastic claims that ‘there are’ actually no ‘objects’ and that ‘structure’ is all ‘there is’ would only partially fit a study of consciousness in relation to its neurophysiological associated phenomena examined in neuroscience and cognitive science nowadays. By ascribing a causal ontological role only to ‘relations’ between ‘objects’ (for instance, brain areas) that generate neurophysiological phenomena related to consciousness and not to these ‘objects’ as well, the OSR would neglect an important part of the empirical study of the neurophysiological phenomena associated with consciousness. Moreover, the OSR would emphasise the ontological ‘What is consciousness?’ question at a moment when neuroscience and cognitive science are not yet prepared to fully answer that question and neither are mathematics or philosophy. That would confound the matter even more right now.

Instead of the OSR, which is the ‘strong’ version of structural realism, I would thus propose its ‘weaker’ version, the epistemic structural realism (ESR). Its more moderate claim—that all we can ‘know’ is the ‘structure of the relations between objects’ and not the objects themselves—could actually support further discussion on neurophysiological phenomena associated with consciousness by concentrating on the epistemological (explanatory) value of discovering these ‘relations’ between ‘objects’ (indeed hard to pinpoint when discussing consciousness as a mental function of the brain). Furthermore, by emphasising the retention of structure across theory change through the structural or mathematical aspects of our theories, the ESR would be again epistemologically relevant for the study of consciousness and its neurophysiological associated phenomena. It would emphasise the ‘What can we know about consciousness?’ question, one which neuroscience and cognitive science could now answer (also with the help of philosophy and mathematics), toward a later (and hopefully not very late) better or even complete ontological explanation of what human consciousness may be (cf. [16,17,18,19,20,21,22,23,24,25,26,27,28,29] for the ‘objects’ vs. ‘structure’ discussion and the differences between the OSR and the ESR).

I would only add now that other specialists also challenge the OSR on the basis of the metaphysical principle of the identity of the indiscernibles, the ESR being more plausible for them [30]. But others argue against the ESR itself (especially against the version that uses Ramsey sentences) [31] and advocate the OSR starting from considerations deriving from the hole argument in general relativity and the status of particles in quantum physics [32]. To briefly explain the first technical term here, Ramsey (or Carnap) sentences are formal logical reconstructions of theoretical propositions that try to separate science from metaphysics. Starting from the distinction between scientific (or ‘real’) questions and metaphysical (or ‘pseudo-‘) questions, in a Ramsey sentence, the so-called ‘observational’ terms replace the ‘non-observable’ theoretical terms. The ‘observational’ terms are to be found in the ‘observation’ or empirical language. This actually translates the ‘kennen’/‘erkennen’ distinction in the German language. I would reject such criticism of the ESR as irrelevant for the present article because I am not proposing the ESR version that uses Ramsey sentences here and I am not trying to thus separate science from metaphysics. As I have already suggested above, I am simply inspired in my work by Quine’s idea that philosophy is not a simple conceptual analysis, but the abstract branch of the empirical sciences [13] and I am trying to highlight a feasible philosophical meta-theory for the empirical study of consciousness and its associated neurophysiological phenomena.

As for the OSR and the hole argument, the latter should be discussed in the context of modern spacetime physics, where it refers to a ‘gauge freedom in general relativity’—the assumption of surplus mathematical structure in general relativity that has no correlate in physical reality. This itself is rather irrelevant for our discussion of consciousness and its neurophysiological associated phenomena since I will take into account studies of consciousness with a bottom up approach: from the physical reality to the later deduced mathematical structures. I thus propose the use of the ESR only as a philosophical meta-theory that would encompass the mathematical structures already discovered by neuroscientists and cognitivists in their experiments. I do not propose an OSR approach that would impose surplus mathematical structures on empirically studied clusters of data on the neurophysiological phenomena associated with consciousness.

I would thus only acknowledge the importance of the concept of structure for every type of inquiry in both science and philosophy when referring to both the recognition and the observation of the nature and stability of relations between various entities. I would also only emphasise that both the principles of empirical sciences and the principles of logics and philosophy are formalised and axiomatised by using an interpretation in order to model reality and create a formal/ theoretical system. And since any structured modeling employs a mathematical framework in order to represent a large variety of models, the ESR would be able to act as a meta-theory that would help bridge various such model types. This would be in tune not only with such structure-based scientific models in general, but also to network neuroscience models in particular. It would take into account the importance of interconnectivity as a fundamental organising principle of the nervous system and would contextualise both various network neuroscience models and the mathematical tools they employ to relate a system’s architecture to its function and dynamics. This would not be an ontological approach—it would not necessarily aim at explaining ‘reality’. It would just aim at explaining our ‘knowledge of reality’ and it would thus be an epistemological approach that would help researchers better understand how interconnected the multiple aspects of the ‘science of consciousness’ can be at this point in time.

This is a written version of an invited talk at the ‘Models of Consciousness’ conference held at the Mathematical Institute of the University of Oxford in September 2019. The considerations on free will (as conscious free choice) were added later. Beyond this introductory section on the ESR vs. OSR, the present article will also contain a section on the possible employment of the ESR as a philosophical framework for the processing of empirical data related to consciousness (and particularly to two scientific models of consciousness) (Section 2) and two more sections offering brief concrete examples of such interdisciplinary methodology: on the one hand, on the possible employment of the ESR as a higher theoretical framework for the processing of empirical data related to free will or conscious free choice (Section 3) and, on the other hand, on its possible employment as a theoretical framework for the processing of empirical data related to AOCs generated by pathologies such as epilepsy (Section 4). These two sections, introduced here just to briefly exemplify my idea of an ESR meta-theoretical framework for the study of consciousness, will be fully developed in a future study.

## 2. The ESR, the Processing of Empirical Data Related to Consciousness and Two Related Scientific Models of Consciousness

Research on the so-called neural correlates of consciousness (NCCs) is often considered a first step in understanding human consciousness. According to David Chalmers, the NCCs are just minimal neural systems whose states can be mapped and this mapping can give some explanations regarding corresponding states of consciousness under specific conditions [33]. Christof Koch also speaks of ‘minimal neural mechanisms jointly sufficient for any specific conscious experience’ [9]. And they both emphasise the ‘causal sufficiency’ of a neural correlate for consciousness. But correlation does not always imply sufficiency and this may be a tricky question that cannot be fully answered since there are also correlates that are not explanatory. The neural explanation is thus limited, as Levine observed when postulating the ‘explanatory gap’ decades ago [34] and as Chalmers also maintained when referring to the ‘hard problem of consciousness’ [35,36]. For this reason, as I have already noted above, in the introductory section, I prefer the term neurophysiological phenomena associated with consciousness to the well known, but still controversial NCCs term.

However, despite the problematic causal import of the neurophysiological phenomena associated with consciousness and even if not all researchers go down to the ‘minimal’ neural systems while some even criticise this approach [37,38], there is still a lot to be discovered about consciousness and its associated neurophysiological phenomena clustered in systematically processed data if we use an interdisciplinary approach facilitated by an ESR meta-theory. This could account for neuroscience and cognitive science data while emphasising the mathematical structure at the level of both primary and processed data and could also account for higher level, philosophical meta-structures and explanations that can be abstracted from the primary and processed data on the neurophysiological phenomena associated with consciousness.

Neural data, computational models and also philosophical analysis can be thus compared and combined in order to identify new principles that connect brain activity to consciousness—be it ‘generic consciousness’ (how the neural properties may explain whether a state is conscious or not) or ‘specific consciousness’ (how the neural properties may explain the particular content of a conscious state). And even unconscious information processing [39,40,41] can be taken into account to further understand consciousness. An ESR meta-theory for the study of consciousness would offer a broad enough paradigm for all these and it would follow the already mentioned path opened many years ago by Quine’s ‘naturalism’ and his methodological critique of traditional philosophy. As Quine asserted, ‘our best theories are our best ‘scientific’ theories’. Consequently, rather than starting from first principles, as in traditional metaphysics, both Quine and his followers advised that philosophers should look at our currently best scientific theories, which may contain (even if only implicitly sometimes) our best account of ‘what exists’, ‘what we know’, and ‘how we know it’ nowadays [42,43]. And this is the approach I am also trying to propose here while supporting an ESR framework of philosophical research for neuroscience.

To further explain the application of an ESR meta-theory and the emphasis on mathematical structures in the study of consciousness and its associated neurophysiological phenomena, I should add here that I actually understand structures as consisting of places that stand in structural relations to each other and mathematical theories as describing such places or positions in structures. This is an approach that would perfectly fit a more theoretical account of the mathematically gathered and processed empirical data on brain and consciousness. Furthermore, since all systems are instantiations of structures and also contain structural properties over and above those that are relevant for the structures they are taken to instantiate, these structural ‘meta-properties’ can ensure the continuity in the shift from one theory to another. 

This continuity, which is obviously one of structure and not one of content [29], would be very suitable for theoretically accounting for the primary and processed data on the neurophysiological phenomena associated with consciousness, which tend to change quite quickly nowadays, but can still be retrieved and integrated via a mathematical structure that accounts for both old and new empirical findings at a higher level and also via an ESR philosophical meta-theory encompassing them all. While emphasising the retention of structure across theory change in this respect, I am also following the path opened by John Worrall, who actually imposed structuralism in the contemporary philosophy of science. I agree here with Worrall’s praise of structural realism—on the one hand, for avoiding pessimistic meta-inductions (by not committing one to believe in the theoretical descriptions of the ‘furniture of the world’) and, on the other hand, for not making the success of science seem miraculous (by committing us to the claim that theoretical structures describe the world over and above their empirical content) [44,45,46].

What I should also explain here is that the ESR meta-theory for neuroscience and cognitive science accounts of consciousness and its associated neurophysiological phenomena can be applied only to those models that are committed to the notions of mental representation and neural representation. While there is still no widely accepted theory on how mental representations get their meaning and there is a huge number of neural properties that may be relevant in explaining mental phenomena in general and consciousness in particular, it is generally accepted that the processing of the neural information provided by the neural sensory systems can be related to the notion of neural representation—even if the definition of the neural representation is itself still problematic [47,48]. Those claiming a strong correlation between the phenomenal content and the neural content would insist on their identity. Those claiming a weaker correlation between the two, would only pose supervenience, which would imply that no change would appear in the phenomenal content without a change in the neural content. 

While taking these into account, one can indeed find a model for the study of consciousness and associated neurophysiological phenomena in neuroscience and cognitive science that perfectly matches the ESR meta-theory and this is the *‘global neuronal workspace’*, proposed by Stanislas Dehaene and his research group and inspired by the cognitive or computational model dubbed global workspace theory (GWT) of consciousness, which was initially proposed by Bernard Baars [49]. Thus, the *‘global neuronal workspace’ model* asserts that a state is conscious when and only when that state or its content are present in the global neuronal workspace and become thus accessible to multiple systems, among which motor system, perception, attention, evaluation, long-term memory, etc. Access should be understood here as a relational notion—a system accesses content from another system if it uses that content in its own computations or processing. And access is also related to brain architecture. It assumes a cortical structure that comprises of workspace neurons with long-range connections between the above-mentioned systems [50,51,52]. Furthermore, the workspace is not a rigid neural structure. On the contrary, it is a rapidly changing neural network. And the model does not attempt to account only for access consciousness, but also for phenomenal consciousness since it considers that a widespread activation of a cortical workspace network is correlated with phenomenal conscious experience [41].

One could think that one would hardly find a model in neuroscience that is more pliable to an ESR philosophical meta-theory committed to relational explanations and retention of structure throughout change than this *‘global neuronal workspace’* model of consciousness. However, a model that could also perfectly match the ESR framework is the *‘integrated information theory’* (or, as formerly also dubbed, the ‘information integration theory’) (IIT) *of consciousness* model proposed by Giulio Tononi. This model employs the notion of *‘integrated information’* (Φ) in order to explain generic consciousness [53,54,55], where Φ is the effective information carried by the parts of the neural system when considering its causal profile. Thus, according to Tononi, a neural system as a whole contains *‘integrated information’* if the effective informational content of the whole is greater than the sum of the informational content of the parts and it is not partitioned. In this case, Φ has a positive value that appears due to the interaction of the parts of the neural system and this positive value of Φ implies that the neural system is conscious (the greater the Φ, the more conscious the neural system). In perfect attunement with the ESR claims, the appropriate connections and interactions are thus more important for consciousness and conscious agency than the amount of neurons activated at a specific moment. The relations and the meta-structure do make the difference for a system to become conscious, that is to obtain a positive value for Φ. Moreover, the mathematical expressions of these relations and structures (or meta-structures) do have a higher explanatory value for consciousness than specific data on specific parts of the neural system.

While only noting here the capacity of an ESR philosophical meta-theory to compare, connect, bridge and possibly help interact these two mathematically expressed models of consciousness in neuroscience and cognitive science, more practical applications of the structuralist principles will be studied in the next two sections: on free will (or conscious free choice) and on the AOCs generated by various pathologies such as epilepsy. The relevance of the ESR for a meta-interpretation of the processing of the related empirical data will be again pointed out.

## 3. The ESR and the Processing of Empirical Data Related to Free Will (or Conscious Free Choice)

I should explain here why a few considerations on free will (or rather conscious free choice) and its own associated neurophysiological phenomena would further help explain the matter of consciousness and its associated neurophysiological phenomena within an ESR meta-theoretical approach. Free will (or free choice—Lat. *liberum arbitrium*—or rather conscious free choice, as I prefer to emphasise its conscious aspect here) is conceived as the capacity of rational agents to choose a particular course of action among various alternatives. As I will show below, conscious free choice employs different cerebral circuits than those employed for automatic action. The study of the interconnectivity and interplay between these changing neural structures and relations during conscious free choice within the philosophical ESR paradigm (which emphasises our knowledge of structural relations between objects rather than of the objects themselves and, at the same time, the retention of structure during change) would mean that both philosophers and neuroscientists could better apprehend the functioning of human consciousness in action. Rather than simply looking at the more or less established brain areas related to consciousness, with established neural functions, during established types of activations related to consciousness (verifying attention, memory, etc.), both philosophers and neuroscientists would study all these during much more complex actions, which would offer both more complex empirical data and more complex theoretical explanations for them. That would certainly require time, higher processing power, and numerous meta-theoretical contextualisations, but would indeed help specialists shed further light on the neurophysiological phenomena associated with consciousness in general as well.

For instance, the topology of structural connectivity seems to be somewhat attuned to support enhanced ignition dynamics—a fast transition from low to high activity that is essential for the emergence of conscious perception and decision making. Moreover, the intrinsic tendency of different regions to become ignited seems to be determined by the specific topological organisation of the structural connectome [56]. But neuroscientists also discovered that brain lesions that disrupt volition or agency occur in many different locations while still remaining within a single brain network that is connected to the anterior cingulate. In a similar manner, lesions that disrupt agency can also appear in other locations, but remain within a network that is connected to the precuneus. And together, these networks may underlie our perception of free will [57]. But this is not enough to understand free will or conscious free choice in its full complexity. As suggested above, numerous scientific and philosophical contextualisations would still be necessary.

Very important for the understanding of the neurophysiological phenomena associated with conscious free choice is, for instance, also the difference between conscious action and automatic response. Recent developments in neuroscience allow for more precise explanations at least on the source of an agent’s actions in as much as they are related to bodily phenomena involved in voluntary action as opposed to those related just to a simple reflex. Thus, while a reflex is an immediate motor response and its form is determined by its specific type of stimulus, the form of a voluntary action is not directly determined by an external identifiable stimulus or is only indirectly determined by it. Furthermore, voluntary action involves the cerebral cortex (and by that, intention and conscious agency) while some of the reflexes involve exclusively the spinal cord. For instance, we cross the street when we do have a reason to do that, not simply whenever we see the green light [58,59].

The idea that automatically stems from this fact is that a voluntary action, as an instance of conscious free choice and agency, can be modelled, interpreted and understood with much more difficulty and in a much more complex manner than a simple reflex. But it would still be important to try to model it since it may be relevant for the manner in which we perceive the relationship between consciousness and its associated neurophysiological phenomena as well. And here the ESR can again help substantially by acting as a philosophical meta-theory for the mathematical modeling of the ‘relations’ between ‘objects’ (and also between ‘actions’ this time) and the establishment of some dynamic structures or patterns for all these. Given the long-accepted philosophical import of the notion of free will (or conscious free choice), the relevance of ESR as a philosophical meta-theory for scientific data cannot but be augmented here.

Unfortunately for this type of research, since voluntary action is not entirely dependent on external stimuli, it is obviously difficult to measure. Regarding such a measurement, the 1983 experiment of Benjamin Libet is generally still brought up when discussing the difficulty of measuring voluntary action and so are also the challenging and later to be also widely challenged conclusions of his experiment: (1) subconscious cerebral processes initiate voluntary actions before the emergence of conscious attention; (2) volitional conscious control does not operate in order to initiate the process of willing, but to control the realisation or, on the contrary, the suprimation of the final motor response initiated by the subconscious processes—conscious volitional control would thus express only a ‘veto’ regarding motor activation [60]. Plainly speaking, according to Libet, even in voluntary actions, the source of action is subconscious, not fully conscious.

Much later, Perez et al. proposed an experiment that was somewhat subtler than that performed by Libet in as much as it was more in tune with possible real-life situations. The goal of this experiment was to separate the act of decision-making from the motor level that would complete the action decided upon [61]. Nonetheless, neither Libet nor Perez or their collaborators seem to have taken into account the context in which the experiment was made—that people intended to participate in the experiment, were informed about it, knew about the results of other subjects’ actions during the experiment, etc. In short, they were ‘conscious’ of the fact that it was an experiment and this was a context that may have had a considerable impact on the ‘readiness potential’ and the conscious/ subconscious control Libet and Perez were talking about. And here the ESR theoretical framework would help by integrating all this information in a wider and subtler pattern of networking ‘relations’ toward a broader and more general approach that may qualify the empirical results and integrate the various mathematical models and that may lead to slightly different or more qualified and qualitatively defined conclusions.

Moreover, as Patrick Haggard explained sometime later, the cerebral circuits involved in voluntary action are a few motor circuits that converge towards the primary motor cortex, which has an executive function in motor commands through the transmission of commands to the spinal cord and the muscles. For this reason, the primary motor cortex area is considered a final common path for voluntary action. It receives impulses from the basal ganglia through the pre-supplementary motor area (preSMA). But the preSMA is included in a larger cognitive-motor circuit, which also consists of the premotor cortex, the cingulate cortex and the frontopolar cortex [58]. Furthermore, a second cortical circuit converging towards the primary motor cortex is involved in the sensory guidance of actions. Within this circuit, the information in the primary sensory areas reaches the parietal cortex, then the premotor cortex and then the primary motor cortex. While using sensory information, this parietal-premotor circuit, guides actions oriented towards a specific object (such as grasping the respective object). And these are more or less fixed relations and structures that describe conscious targeted choice and action.

But research done on neurons from the lateral intraparietal area (LIP) in primates indicate that these neurons encode the choice done by the animal when it is confronted with two alternatives that have the same reward value. According to this encoding, when immediate action is necessary, the parieto-motor circuit arbitrates between alternative actions while in the absence of emergency, the basal ganglia-preSMA circuit is primarily involved in the initiation of action. The two cerebral circuits seem to be involved in decisions of different types and initiations of voluntary actions of different types, which are both relevant for the human free choice [62]. Arbitrating among cerebral circuits and relating differently to each of them depending on the given context elicit again a type of neuroscience research that is pliable to an ESR philosophical meta-theory that can offer general and theoretical explanations to a large array of mathematically expressed, but still fluctuating empirical data on changing, but still resilient connections between brain areas. And all this would be relevant for the study of consciousness in general during different types of actions.

A neuroscientist could thus conclude from the above information that voluntary actions appear when: (1) the routine processing of the stimuli does not furnish sufficient information in order to determine an answer; (2) when new reasons for action appear [63]. Many neuroscientists would thus consider free will or conscious free choice a simple illusion. For instance, Wegner asserts that the human mind postulates a causal way from the conscious intention to act towards the action itself in order to explain the temporal correlation between the two events; but it may happen that the correlation appears due to the fact that both the conscious intention and the action have a common cause, which is the neural readiness for action. Thus, both events could actually be the consequences of a previous cerebral activity [64] that is not necessarily conscious. Likewise, Haggard and Chambon postulate a sixth sense, the sense of agency, and insist that voluntary actions are accompanied by specific subjective experiences such as the experience of intention and the experience of being an agent (the idea that a person’s voluntary actions generate specific events in the outer world in a conscious way) [59].

In this respect and for the moment, if they ever accept free will or conscious free choice, neuroscientists prefer a rather pragmatic conception of it. They insist that experimental data suggest that the sense of agency depends on prospective processes and does not represent a retrospective confabulation. The brain represents prospectively the result of an action before the action is produced. If the subjects are exposed to priming subliminal stimuli for a short time before the action is performed and these subliminal stimuli are either compatible or incompatible with the action (through the consequences produced by the action), the subject can report a more intense sense of agency (namely the idea that they can control the results of the action) when there is a correspondence between the subliminal stimulus and the task stimulus presented [65]. Studies of social psychology also emphasise that persons being exposed to the idea that free will or free conscious choice does not exist are more predisposed to asocial behaviour such as cheating and aggressivity or their prosocial and altruistic behaviour is reduced. The individual lack of trust in free conscious choice seems to reduce the capacity of the individual to activate his/ her self-control. Self-control is demanding and energy-consuming and the denial of free conscious choice reduces the disponibility to invest this energy in an action that is thus considered ineffective [66,67].

To all these issues, an ESR meta-theoretical approach would be able to offer some fine-tuned contexts that would help make the matter a bit more subtle. Maybe, beyond the neuroscience experiments, that provide a context somewhat artificially limited in terms of time and types of actions, we should also take into account the wider context of those types of actions even more? Maybe the subconsciously generated action or the readiness potential actually follow a previous or higher-order conscious command of the subject of the experiment—such as his/ her conscious acceptance of his/ her participation in the experiment and of the environment and requirements it poses? These remain, of course, questions to be answered in future by us all—neuroscientists, cognitivists and philosophers altogether—possibly within and ESR meta-theoretical context, in which mathematically expressed structures and interrelations could still be maintained at a higher-order and more general level of discussion despite the changes that may occur in the latest empirical data. But, due to considerations of limited time and space in this article, this will remain, for the moment, just a proposal for a future fully developed study.

## 4. The ESR and the Processing of Empirical Data Related to Alterations of Consciousness (AOCs) Generated by Various Pathologies—The Case of Epilepsy

As noted above, according to the global workspace theory (GWT), conscious processing is the result of neuronal activity that is structured and coherent between widely distributed brain regions, the most important elements in this processing being the fronto-parietal associative cortex. And pathologies that generate AOCs can offer very useful information in this respect. For instance, a transition from conscious to non-conscious states during epileptic seizures seems to be caused by sudden non-linear changes of the level of coherence within the neuronal space. The study of epileptic seizures can thus even demonstrate the validity of the global workspace theory (GWT). The sudden AOCs often occurring during epileptic seizures have been proved to be simultaneous with non-linear increases of neural synchrony within distant cortico-cortical and cortico-thalamic networks. Within the GWT model, such an excessive synchrony could thus prevent the network to reach the levels of complexity and differentiation that are necessary for conscious representations. On the basis of such research, neuroscientists would thus be able to better specify the minimum and maximum neural coherence related to conscious processing [68].

To explain the pathology a bit more, epilepsy can be characterized by a recurrent and temporary brain dysfunction generated by discharges of interconnected groups of neurons that sometimes create large-scale brain networks. For instance, in absence epilepsy (AS), the analysis of dynamic changes of anti-correlation between the thalamus and the so-called default mode network (DMN) can be associated with an inhibitory effect of seizures on the default mode network, which gradually stops functioning after seizure onset. Complex adaptive reconfigurations of the large-scale functional connectome also appear [69].

There are actually numerous research groups working on epilepsy that target the role of the DMN in losing and re-gaining consciousness. They generally study the dynamics of conscious states in respect to epileptic activity as an interaction between the two functional networks: the physiological (DMN) and the pathological (the epileptome). In this respect, there are two main theories: ‘the network inhibition’ hypothesis, asserting that there is an indirect inhibition of the DMN via the profound diencephalic structures (thalamus) [70], and the ‘diminished workspace’ hypothesis, asserting that during the seizure more and more hubs of the DMN connectome are recruited by the epileptome [71]. The mechanism by which the reverse happens—the sudden or gradual regaining of consciousness in the postictal phase—is still insufficiently studied, but experts have generally agreed that each of the five types of AOCs that occur during epileptic seizures (auras with illusions or hallucinations, dyscognitive seizures, epileptic delirium, dialeptic seizures, and epileptic coma) have a particular manner in which they impact the DMN and thus the subjective conscious experience of the patient. And this research is also pliable for an ESR philosophical interpretation, in which mathematically collected and processed empirical data can be made relevant also at a higher, meta-theoretical level, that would make them generally relevant for consciousness and its neurophysiological associated phenomena.

For instance, during the last years, in order to offer the best possible chances to the patients during surgery, losing and re-gaining consciousness due to epilepsy have been thoroughly studied during the presurgical stage through intracranial electroencephalograpy (iEEG). Such explorations offer the specialists very complex and diverse empirical data that need to be processed and interpreted in the context of intricate brain mapping and connectomics analysis. And the ESR could again offer here a subtle theoretical framework that would be in tune with the neuroscientists’ attempt to process and organise multiple-channel iEEG signals and concentrate on the relationships between various brain areas and on the relevance and the resilience of these relationships for both the healthy and the pathological brain.

Some research groups study the connectivity relationship between these channels by representing it as a matrix for each time slot while the matrices are then regarded as points on a Riemannian manifold. Through this method, the similarity can be measured by the geodesic distance on the manifold, even if the signals are related to an ictal process that invloves continuous changes of information propagation. The Riemannian method offers thus the possibility of figuring out the brain network dynamics by clustering methods that can better localize the seizure onset zone (SOZ) [72]. And this is yet one more example for the ways mathematical structures can help in the processing of complex data and, automatically, for the ways the ESR as a meta-theoretical framework can help in understanding and interpreting them at a higher level for a better study not only of the pathological AOCs, but also of human consciousness in general.

I should add now some clarifications on the technical terms used above that are also relevant for my attempt to propose the ESR as a meta-theoretical framework for the empirical and mathematical studies of consciousness. For instance, the word ictal, of Latin origin (blow, stroke), refers to a physiological state or event such as a seizure, stroke, or headache. In electroencephalography (EEG), the recording during seizure is said to be ‘ictal’ and it refers to a complex network of cerebral interconnected hubs. Likewise, the epileptic focus (seizure onset zone—SOZ) localization is obtained via brain network analysis and brain network connectivity and plays an important part in the computer-aided automatic localization of the SOZ. But how the specialists interpret and understand this brain network connectivity and its personal specific implications is a much more complex matter, which would greatly benefit from the ESR meta-theoretical focus on ‘relations’ between objects and retained mathematical structures through change (or variation) of both empirical data and theoretical interpretations.

Furthermore and finally, neural stimulation during the presurgical stages is also very important both for collecting empirical data on the pathological brain that are necessary for the treatment of epilepsy and for a better understanding of AOCs and of consciousness in general. Although there are limited opportunities to manipulate human brain activity in a targeted way, the recent use of transcranial magnetic stimulation to activate or suppress neural activity has provided extremely useful data for understanding human consciousness, even if such interventions are still not fine-grained enough to perfectly locate an explanatory correlate for specific conscious contents.

Specific intracranial stimulation paradigms are generally used for presurgical evaluations. These are either low frequency subclinical stimulation protocols (single pulse electrical stimulation (SPES) and 1 Hz), used to perform effective connectivity studies, or high frequency clinical protocols (50 Hz), used to obtain a functional mapping of different brain areas with various behavioural effects. The post-processing of the raw intracranial recording and a Fourier analysis can quantify the amount of activation in gamma (30–90 Hz) power and coherence, which is a direct reflection of the activity in local neural circuits. The intracranial gamma signal during the different activations related to various types of ictal AOCs and also the inter-ictal connectivity are analysed in order to describe the functional networks behind the complex dynamics created by the pathological condition of the patient. What has already been demonstrated is that the seizure onset zone (SOZ) connectivity (the connectivity of the epileptic focus area) is not a static, but a dynamic concept, engaging in a variety of network configurations that can be accurately described in a personalized manner [73]. And this could again match a relations-oriented ESR meta-theoretical framework that asserts the endurance of mathematical structures for such dynamic patterns. But this section itself is, like the previous one, only a short proposal and illustration of possible applications of the ESR as a meta-theoretical framework for the study of consciousness and it needs to be further developed in a much more complex and extensive study later on.
